# Acute acalculous cholecystitis complicated by infectious mononucleosis caused by cytomegalovirus

**DOI:** 10.1002/ccr3.8771

**Published:** 2024-04-17

**Authors:** Noriko Ide, Risa Hirata, So Motomura, Masaki Tago

**Affiliations:** ^1^ Department of General Medicine Saga University Hospital Saga Japan

**Keywords:** acalculous cholecystitis, cytomegalovirus, epigastric pain, infectious mononucleosis

## Abstract

**Key Clinical Message:**

When seeing patients who present with atypical lymphocytes and abdominal pain without accompanying symptoms of pharyngitis or lymphadenopathy, acalculous cholecystitis caused by CMV infection should be considered as a differential diagnosis.

**Abstract:**

A teenage man presented with a fever and epigastric pain. The patient tested positive for cytomegalovirus IgG and IgM. Abdominal ultrasonography and contrast‐enhanced CT revealed hepatosplenomegaly and gallbladder wall thickening. MRI did not identify gallstones or tumorous lesions. He was diagnosed with infectious mononucleosis and acalculous cholecystitis caused by cytomegalovirus.

## 1 CASE

**FIGURE 1:**
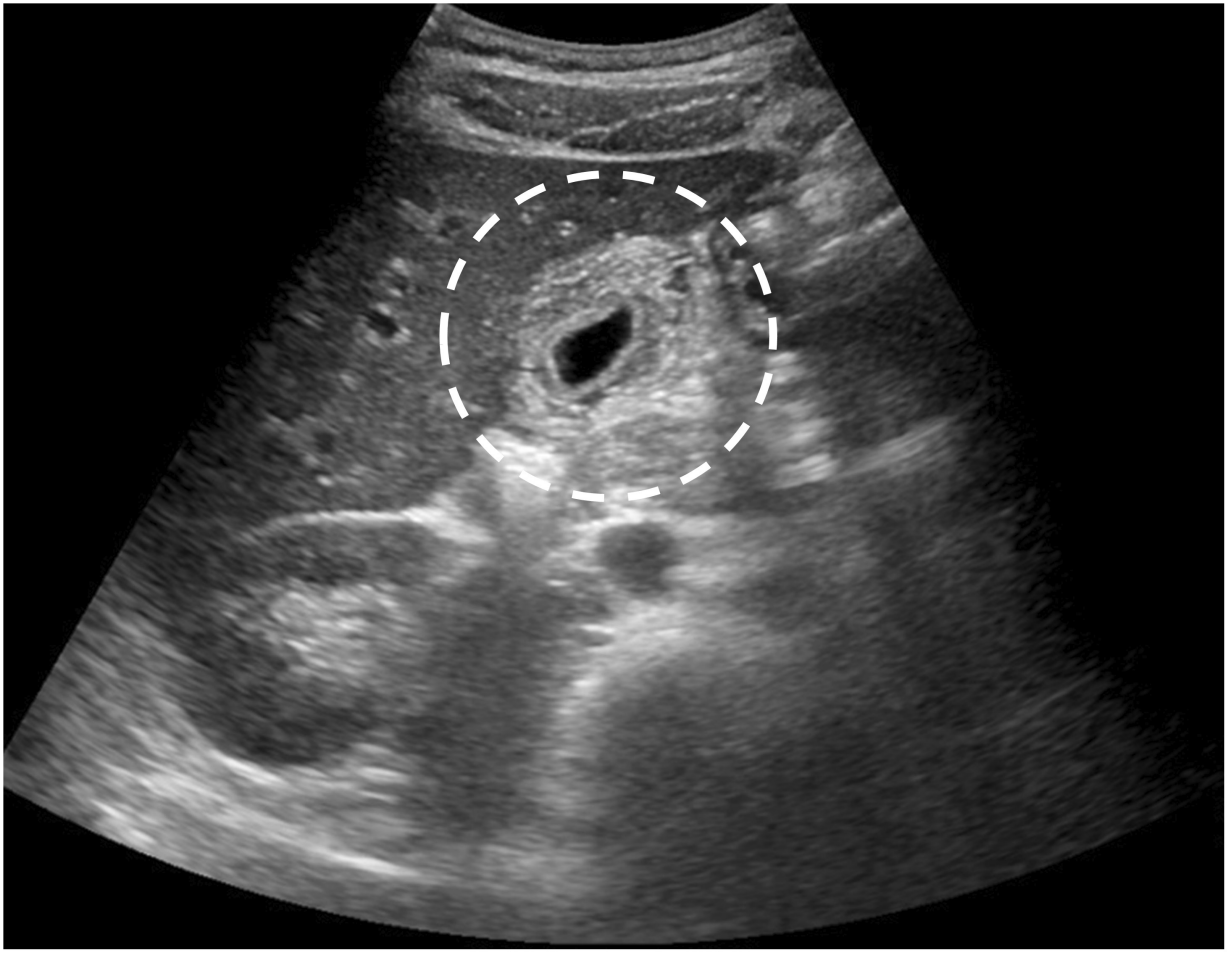
Abdominal ultrasonography. The gallbladder wall is fully thickened around the circumference (dashed circle).

**FIGURE 2:**
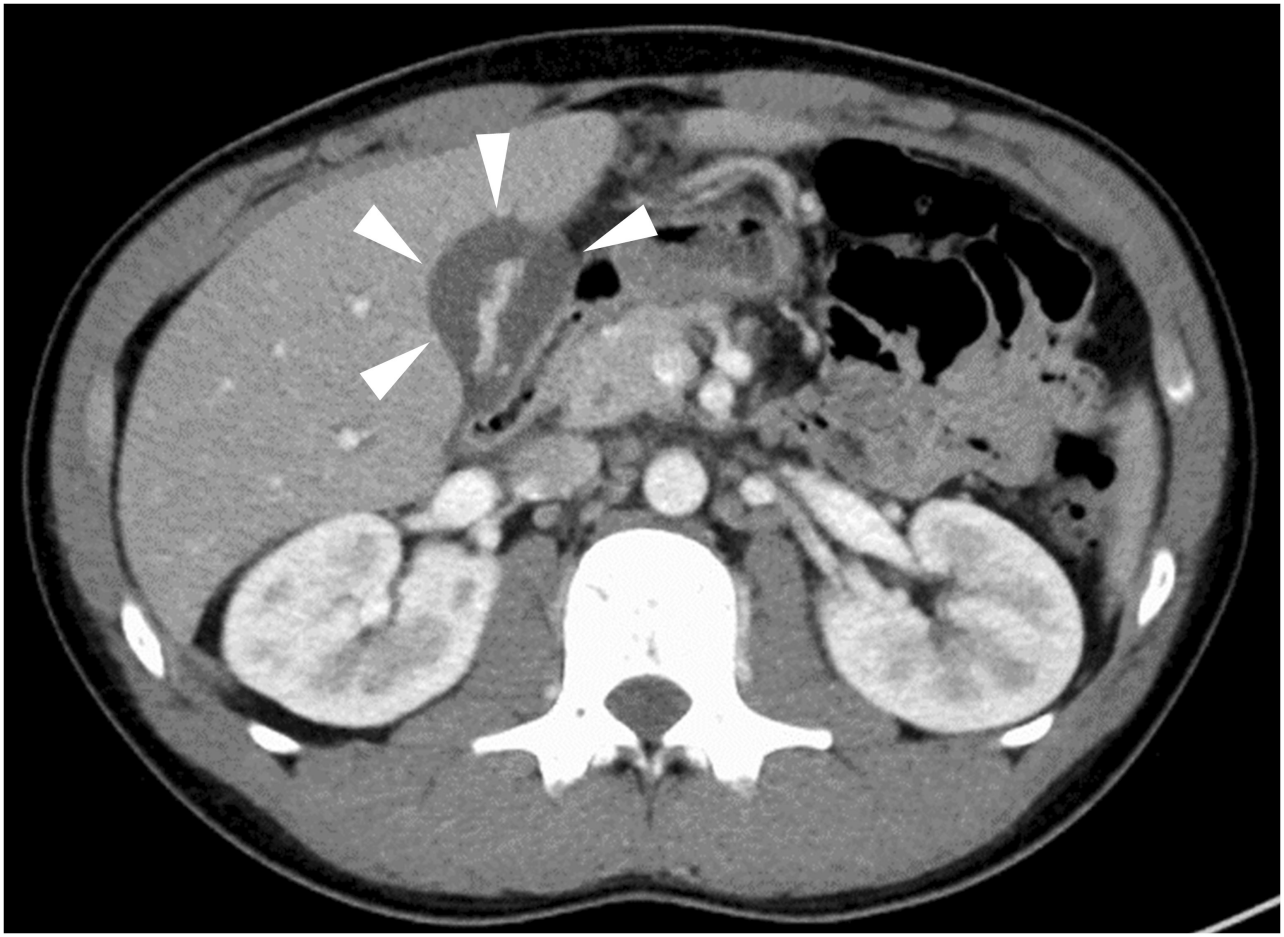
Abdominal contrast‐enhanced CT (axial view). Edematous wall thickening is shown (arrowheads).

A teenage man without any medical history presented with fatigue, cough, and rhinorrhea on Day 0. He developed a fever on Day 10, followed by epigastric pain and loss of appetite on Day 12. Consequently, he visited our hospital on Day 15. Upon admission, the patient was alert with a body temperature of 38.0°C, blood pressure of 121/78 mmHg, and a heart rate of 108 beats per minute. Physical examination revealed no redness of the throat or swollen neck lymph nodes. However, hepatomegaly and tapping pain from the epigastric region to the right costal region were observed. Blood analysis revealed a white blood cell count of 9.1 × 10^9^/L, atypical lymphocytes of 21.5%, platelet count of 33 × 10^9^/L, C‐reactive protein level of 148 nmol/L, aspartate aminotransferase level of 3616 nkat/L, alanine aminotransferase level of 5766 nkat/L, gamma‐glutamyltransferase level of 6216 nkat/L, alkaline phosphatase level of 4550 nkat/L, and lactate dehydrogenase level of 10,100 nkat/L. The patient tested positive for cytomegalovirus (CMV) IgG at 47.5 AU/mL and CMV IgM at 24.2 AU/mL, leading to a diagnosis of infectious mononucleosis caused by CMV. Furthermore, abdominal ultrasonography and contrast‐enhanced computed tomography revealed hepatosplenomegaly and marked gallbladder wall thickening (Figures 1 and 2). Magnetic resonance cholangiopancreatography did not identify gallstones or tumorous lesions in the gallbladder or bile ducts, leading to a diagnosis of acalculous cholecystitis caused by CMV infection. The patient received conservative treatment without antibiotics during his hospitalization, and his abdominal findings improved. The patient was discharged home on Day 23. On Day 33, a follow‐up abdominal ultrasonography showed the disappearance of the gallbladder wall thickening.

The most common cause of infectious mononucleosis is Epstein–Barr virus (EBV) infection, although it can also be caused by CMV, toxoplasmosis, and human herpesvirus‐6. CMV‐induced infectious mononucleosis presents with nonspecific symptoms, making a diagnosis challenging. Compared with EBV‐induced infectious mononucleosis, CMV‐induced cases often exhibit less prominent signs of pharyngitis and lymphadenopathy, with predominant systemic symptoms including fatigue and fever.^1^ Furthermore, cases of acalculous cholecystitis complicating infectious mononucleosis in non‐immunocompromised individuals, as in the present case, are extremely rare, and to the best of our knowledge, there have been very few published case reports.^2^ To promptly diagnose such cases, it is crucial to recognize that even in non‐immunocompromised individuals, acalculous cholecystitis can occur concurrently with infectious mononucleosis caused by CMV. Acalculous cholecystitis is more prone to occur in immunocompromised or septic patients, and acalculous cholecystitis is caused by CMV, EBV, Hepatitis A virus, or Hepatitis B virus infection. Acalculous cholecystitis in immunocompromised individuals tends to worsen, requiring the administration of antibiotics as well as surgical interventions and the long‐term use of antiviral drugs.^3^ However, acalculous cholecystitis caused by CMV in non‐immunocompromised individuals often resolves with conservative treatment alone, without the need for antibiotics.

In conclusion, when seeing patients, even non‐immunocompromised individuals, who present with atypical lymphocytes and abdominal pain without accompanying symptoms of pharyngitis or lymphadenopathy, acalculous cholecystitis caused by CMV infection should be considered as a differential diagnosis, and virological testing should be conducted.

## AUTHOR CONTRIBUTIONS

**Noriko Ide:** Conceptualization; investigation; writing – original draft. **Risa Hirata:** Conceptualization; investigation; writing – original draft. **So Motomura:** Investigation; writing – review and editing. **Masaki Tago:** Conceptualization; investigation; supervision; writing – original draft; writing – review and editing.

## FUNDING INFORMATION

There is no funding for this article.

## CONFLICT OF INTEREST STATEMENT

The authors state that they have no conflict of interest.

## ETHICAL APPROVAL

This manuscript conforms to the provisions of the Declaration of Helsinki in 1995 (as revised in Brazil 2013).

## CONSENT

Written informed consent was obtained from the patient to publish this report in accordance with the journal's patient consent policy.

## Data Availability

The data that support the findings of this study are available from the corresponding author upon reasonable request.
